# Radiographic and clinical outcomes of traumatic subtrochanteric femur fracture fixation and reduction methods

**DOI:** 10.1007/s00590-025-04478-z

**Published:** 2025-08-18

**Authors:** Micaela White, Rebeka Dejenie, Shannon Tse, Branden Brooks, Michelle Scott, George Chavez, Lydia McKeithan, Aziz Saade, Samuel K. Simister, Sean T. Campbell, Gillian Soles, Ellen Fitzpatrick, Mark Lee, Augustine M. Saiz

**Affiliations:** 1 University of California Davis School of Medicine, Sacramento, USA; 2 Department of Orthopaedic Surgery, University of California, Davis, Sacramento, USA

**Keywords:** Keywords, Subtrochanteric femur fractures, Trauma, Fixation, Radiographic analysis

## Abstract

**Purpose:**

Different reduction methods and implant choices for subtrochanteric femur fractures with varying fracture patterns have been described. This study aimed to assess the relationship of fracture patterns, reduction methods, and implant selection with radiographic and clinical outcomes.

**Methods:**

A retrospective review of patients aged ≥ 18 with traumatic subtrochanteric fractures between 2016 and 2023 at a Level-1 trauma center was conducted. Patient, surgical, and outcome characteristics were collected. Femoral neck-shaft angle , maximum sagittal cortical displacement, and modified radiographic union score of the tibia (mRUST) adapted to the proximal femur were measured on post-operative radiographs.

**Results:**

Among 64 patients (mean age 56.1, 70.3% male) with one patient with bilateral injuries, 54 received an intramedullary nail (IMN), 8 a plate, and 3 a nail-plate combination. No differences were observed in AO/OTA fracture patterns between groups. Open reduction was performed in 35 cases and closed or percutaneous reduction in 30 cases. Closed reductions had significantly shorter surgery durations and decreased estimated blood loss (EBL) compared to open reductions (*p* = 0.028 and *p* = 0.049, respectively). Radiographically, maximal cortical displacement on lateral imaging was significantly increased in closed or percutaneously reduced fractures compared to open reductions (*p* = 0.025). IMN fixation was utilized in patients with higher BMI (*p* = 0.017). IMN fixation resulted in increased maximal cortical displacement on lateral imaging compared to plate and combination nail-plate fixations (*p* = 0.041). No differences in mRUST at multiple time points were observed in reduction method or implant type. Our average length of follow-up was 132 days. Post-operatively, no differences existed in pain, independent ambulation, complication or revision rates between implants or reduction methods.

**Conclusion:**

Subtrochanteric fractures treated with open reduction and presence of a plate in the fixation can improve sagittal alignment. Closed/percutaneous reduction decreases surgery duration and estimated blood loss. Regardless of reduction type and implant, adequate reduction, high union rates and successful outcomes are possible. Anatomic reduction and stable fixation are essential in surgical treatment, regardless of approach or implant choice.

## Introduction

Subtrochanteric femur fractures often require surgical stabilization. These fractures are defined as fractures that occur within 5 cm of the lesser trochanter of the femur [1]. They are frequently caused by low energy mechanisms, such as ground level falls in elderly people and high energy mechanisms in younger people [1] As with any fracture treatment in orthopedic trauma, anatomic reduction and stable fixation is paramount for patient outcomes. However, methods of reduction and implant selection can vary based on patient and fracture patterns.

A gap exists in the literature concerning the comparison between reduction methods, implant selection, radiographic alignment, clinical outcomes, and complications rates across a wide spectrum of patients and treatment modalities. This study therefore aims to bridge this gap. Drawing from prior research, we hypothesized that closed and percutaneous reduction would yield better healing with reduced post-operative wound complications, while open reduction would provide better anatomical alignment and thus, better radiographic outcomes.

## Methods

Following Institutional Review Board approval, a retrospective cohort study investigating patients with subtrochanteric femur fractures treated operatively between September 2016 and September 2023 was performed. Inclusion criteria encompassed adult patients over the age of 18 years with primary traumatic subtrochanteric femur fractures. Patients were excluded if they had cancer with bone metastases. Patients were also excluded if they were undergoing a prophylactic nailing, secondary, or staged procedure. The following patient demographics were collected by retrospective chart review: age, sex, BMI, presence of comorbidities/past medical history, tobacco smoking history, preoperative ambulation status, or ASA classification. Injury radiographs were analyzed for confirmation of subtrochanteric femur fracture and were defined using AO/OTA and Russell-Taylor Classifications. Chart review was conducted to gather demographic data, injury characteristics, and post-operative clinical outcomes.

Radiographs were analyzed separately, and measurements were performed on Visage 7 (Visage Imaging Inc., San Diego, CA, USA). The femoral neck-shaft angle in degrees bilaterally was defined as the angle between a line drawn through the distal fragment of the femoral shaft and a line drawn in the proximal segment centered through the femoral neck perpendicular to the femoral head. Maximum cortical displacement in millimeters on anterior/posterior (AP) and lateral X-rays were defined as the maximum distance between the medial or lateral cortices on the distal and proximal fragments. Angulation at the fracture site in degrees was measured on the AP and lateral imaging defined as the angle between lines drawn centered in the proximal and distal segments. The presence of neutral, varus, or valgus was analyzed from the AP image. The presence of neutral, flexion, or extension was analyzed from the lateral image. The modified Radiological Union Scale for Tibia (mRUST) was used to assess progress to osseous union and assign a numerical assessment 1–4 of fracture healing at 6 weeks, 3 months, 6 months, and 1 year as adapted for the femur [2]. Score of 1 represents no callus, 2 has callus present, 3 has bridging callus, and 4 is remodeled and no visible fracture line. Each individual cortical score (anterior, posterior, medial, and lateral) is added together to yield the mRUST, with a minimum of 4 and maximum of 16. Lower scores indicate poor fracture healing, higher scores indicate good fracture healing and remodeling [2]. This scoring system has been validated as a useful tool in determining osseous union in femur fractures as well [2–4]. The clinical outcomes of interest investigated in this study were hardware failure, revision surgery, non-union, wound complications, clinical follow-up days, pain at follow-up, and independent ambulation.

Unpaired t-tests, Welch’s ANOVA, and Chi-squared statistics compared clinical and radiographic data between open versus closed or percutaneous reduction and implant types. *p* values of < 0.05 were considered significant. All statistical analysis was performed using SPSS (IBM, Chicago, IL, USA).

## Results

Sixty-four patients underwent surgery for subtrochanteric femur fractures at our institution during the study period. The mean age at surgery was 56.1 ± 23.3, and mean BMI was 28.8 ± 8.9. 70.3% of patients were male. One patient underwent surgery for bilateral fractures for a total of 65 fractures—54 received an intramedullary nail (IMN), 8 a plate, and 3 a nail-plate combination (NPC). Of those, open reduction occurred in 35 cases, closed in 8 cases, and percutaneous in 22. Closed and percutaneous reductions were combined for the purposes of our analysis for a total of 30 cases compared to 35 open reductions. Mean follow-up was 132.2 ± 202.8 days. There were no significant differences in reduction method regarding patient demographics, injury variables, including mechanism of injury, open fracture status, or presence of polytrauma or head injury (Table 1).

**Table 1:** Demographics based on reduction methods

	Closed reduction	Open reduction	Total	*P* value
Male	18	28	46	0.107
Female	12	7	19	
			65	
Mean age	56.40 ± 23.30	55.74 ± 23.67		0.911
Mean BMI	29.74 ± 9.77	27.87 ± 8.04		0.432
No significant PMHx	12	19	31	0.205
Significant PMHx	18	15	33	
			64	
Non-smoker	14	17	31	0.906
Smoker^a^	14	16	30	
			61^b^	
Pre-Op independent Ambulator	22	25	47	0.794
Pre-op assistive device used	6	8	14	
			61^c^	
Ground level fall	12	15	27	0.669
High energy fall	2	3	5	
Motor vehicle collision	6	8	14	
Motor cycle collision	2	2	4	
Pedestrian vs automobile	2	2	4	
Assault/crush injury	0	2	2	
Gunshot wound	2	2	4	
Bike vs Automobile	1	1	2	
Chronic stress fracture	2	0	2	
	29	35	64	
Closed fracture	28	32	60	0.773
Open fracture	2	3	5	
	30	35	65	
Isolated Trauma	20	23	43	0.934
Polytrauma	10	11	21	
	30	34	64	
No head injury	26	28	54	0.634
Head injury	4	6	10	
	30	34	64	

^a^Smoker defined as current or former use

^b^Missing tobacco history information for 3 patients

^c^Missing ambulation status pre-op on 3 patients

IMN fixation was performed in 30 closed or percutaneous cases and 24 open cases. Open reduction was the only method for the 8 plate fixation cases and the 3 NPC fixations. Plates were only used in male patients and in patients without presence of significant past medical history (*p* = 0.047 and *p* = 0.002, respectively). IMN fixation was utilized more frequently in patients with higher BMI when comparing the mean BMIs of plate and combination fixations (*p* = 0.017). Regarding other demographic information, there were no statistically significant differences in implant choice (Table 2).

**Table 2:** Demographics based on implant choices

	IMN	Plate	NPC	Total	*p* value
Male	36	8	2	46	0.047
Female	18	0	1	19	
	54	8	3	65	
Mean Age	56.78 ± 24.20	45.63 ± 17.10	70.67 ± 9.30		0.058
Mean BMI	29.63 ± 9.25	23.14 ± 2.30	23.69 ± 2.48		0.017
No significant PMHx	22	8	1	31	0.002
Significant PMHx	31	0	2	33	
	53	8	3	64	
Non-smoker	26	4	1	31	0.817
Smoker	24	4	2	30	
	50^a^	8	3	61	
Pre-Op independent Ambulator	39	7	1	47	0.204
Pre-Op assistive device used	11	1	2	14	
	50^b^	8	3	61	
Ground level fall	22	2	3	27	0.599
High energy fall	4	1	0	5	
Motor vehicle collision	13	1	0	14	
Motor cycle collision	2	2	0	4	
Pedestrian vs Automobile	3	1	0	4	
Assault/crush injury	1	1	0	2	
Gunshot wound	4	0	0	4	
Bike vs automobile	2	0	0	2	
Chronic stress fracture	2	0	0	2	
	53	8	3	64	
Closed fracture	49	8	3	60	0.380
Open fracture	5	0	0	5	
	54	8	3	65	
Isolated Trauma	35	5	3	43	0.287
Polytrauma	18	3	0	21	
	53	8	3	64	
No head injury	44	7	3	54	0.561
Head injury	9	1	0	10	
	53	8	3	64	

^a^Missing tobacco history for 3 IMN patients

^b^Missing preoperative ambulation status for 3 IMN patients

Intra-operatively, we compared reduction methods and implant choices investigating duration of surgery and estimated blood loss. Percutaneous or closed reduction surgeries yielded significantly decreased surgical durations and estimated blood loss compared to open reduction surgeries (*p* = 0.028 and *p* = 0.049, respectively). Implant choice did not statistically significantly impact duration of surgery or estimated blood loss (*p* = 0.111 and *p* = 0.079, respectively).

Differences were noted in radiographic data points of interest for reduction method and implant choice (Table 3). Maximum cortical displacement on lateral imaging was significantly increased in closed or percutaneously reduced fractures compared to open reductions (p = 0.025). Other radiographic outcomes investigated in this analysis were not statistically different in closed or percutaneous reductions compared to open reductions. There were no significant differences in mRUST scores at 6 weeks, 3 months, 6 months, or 1 year comparing reduction types. Radiographically, maximum cortical displacement on lateral imaging was significantly increased in nail fixations compared to plate and combination fixations (*p* = 0.041). All other radiographic imaging points were not statistically significant when comparing implant choices. Notably, there was insufficient follow-up for the different types of implants to perform statistical testing on mRUST data at 1 year.

**Table 3:** Radiographic and clinical follow-up data based on reduction method and implant choice

	Closed reduction	Open reduction	*p* value	IMN	Plate	NPC	*p* value
Contralateral femoral neck-shaft angle (mm)	134.41 ± 6.27	133.85 ± 4.65	0.694	133.93 ± 5.88	135.75 ± 2.66	132.73 ± 2.51	0.260
Femoral neck-shaft angle (mm)	134.24 ± 6.41	134.72 ± 5.48	0.750	134.28 ± 5.92	137.00 ± 5.29	130.30 ± 6.65	0.467
Maximum cortical displacement on AP	6.76 ± 11.14	4.69 ± 5.32	0.336	5.82 ± 8.79	3.44 ± 6.73	7.73 ± 5.90	0.605
Maximum cortical displacement on lateral	6.36 ± 6.47	3.10 ± 3.72	0.025	5.12 ± 5.67	1.71 ± 1.25	2.17 ± 2.93	0.041
Neutral on AP	26	32	0.085	48	8	2	0.454
Varus on AP	4	1		4	0	1	
Valgus on AP	0	2		2		0	
	30	35		54	8	3	
Neutral on lateral	30	33	0.274	53	7	3	0.333
Flexion on lateral	0	1		0	1	0	
Extension on lateral	0	1		1	0	0	
	30	35		54	8	3	
mRUST at 6 weeks	8.75 ± 3.61	7.50 ± 2.53	0.288	8.04 ± 3.34	10.00 ± 2.00	7.00 ± 1.41	0.338
mRUST at 3 months	11.09 ± 3.24	11.00 ± 2.65	0.943	10.94 ± 3.00	13.50 ± 2.12	9.50 ± 0.71	0.204
mRUST at 6 months	13.00 ± 1.83	12.90 ± 2.28	0.925	12.73 ± 2.15	13.25 ± 2.50	13.50 ± 0.71	0.691
mRUST at 1 year	15.60 ± 0.894	14.50 ± 3.00	0.456				
Clinical follow-up (days)	142.93 ± 211.15	123.00 ± 198.07	0.696	139.43 ± 219.76	94.63 ± 84.52	102.33 ± 59.01	0.581
No hardware failure	29	32	0.368	52	6	3	0.139
Hardware failure	1	3		2	2	0	
	30	35		54	8	3	
No revision surgery	29	33	0.644	53	6	3	0.069
Revision surgery	1	2		1	2	0	
	30	35		54	8	3	
Union	28	34	0.464	52	7	3	0.555
Non-union	2	1		2	1	0	
	30	35		54	8	3	
No wound complication	30	34	0.263	53	8	3	0.829
Wound complication	0	1		1	0	0	
	30	35		54	8	3	
No Pain	9	12	0.606	19	2	0	0.209
Pain^a^	17	17		27	4	3	
	26	29		46	6	3	
Post-Op independent Ambulator^b^	14	19	0.374	26	4	3	0.214
Post-op assistive device used	11	9		18	2	0	
	25	28		44	6	3	

^a^Pain information missing from 9 patients

^b^Post-operative ambulation status missing from 11 patients

Regarding clinical follow-up data, reduction type or implant choice did not significantly affect outcomes (Table 3).

## Discussion

Our study compared reduction method and implant choice in the treatment of subtrochanteric femur fractures and the potential effects on radiographic and clinical outcome data. Of note, reduction method and implant choices do have associations with demographics, radiographic outcomes, and clinical outcomes. Specifically, regarding reduction methods, closed/percutaneous reduction yielded shorter surgery duration and decreased blood loss during surgery compared to open reduction. This aligns with previously published research. Panteli et al. found increased surgical duration and need for transfusion within 48 h post-operatively in open reductions in fixation of subtrochanteric femur fractures [5]. Tahir et al. investigated 398 patients who underwent intramedullary nailing for femoral shaft fracture and found operative time to be significantly longer and estimated blood loss to be significantly increased in open reduction operations [6]. This study is less relevant to our study given it includes femoral shaft fractures as opposed to subtrochanteric femur fractures. However, implant choice did not impact these findings which is contradictory to other published research. A systemic review and meta-analysis of 11 studies performed by Xie et al. demonstrated statistically significant shorter operative time and decreased estimated blood loss in intramedullary fixation compared to extramedullary fixation in subtrochanteric femur fractures [7].

The treatment algorithm for subtrochanteric femur fractures has multiple layers. Previous research examining reduction methods for the treatment of subtrochanteric femur fractures has produced varied findings. For instance, Knauf reported that open reduction resulted in statistically significant differences in mobility seven days post-operation [8]. Yao, on the other hand, investigated closed versus limited open reduction with intramedullary nail (IMN) fixation, noting minimal operative revisions and no occurrences of internal fixation failures, fracture displacements, or non-unions [9]. Notably, neither study directly compared nailing versus plating in the treatment of subtrochanteric femur fractures. While existing literature includes comparisons of nailing versus plating outcomes, these analyses often encompass both trochanteric and subtrochanteric fractures, limiting their applicability to isolated subtrochanteric fractures [10,11]. Sensoz and Mirbolook et al. both conducted studies evaluating intramedullary versus plating only in subtrochanteric fractures and found no differences in clinical outcomes such as non-union and complication rates, similar to our study [12,13]. However, these studies did not review radiographic findings which makes our study unique and novel. Additionally, studies such as those by Beingessner focus on post-operative outcomes of either nailing or plating in subtrochanteric fractures [14]. These studies collectively underscore the complexities inherent in selecting the most suitable reduction method and implant type for subtrochanteric femur fractures. They highlight the imperative for further research to facilitate informed clinical decision-making effectively.

Reduction method and implant choice did not have any statistically significant impact on clinical outcome data. Overall, there is conflicting published literature in this area. Panteli et al. found an increase in superficial infection in open reductions, which our study did not find [5]. Our study does align with other results of this study though in that there was not an increase in non-union depending on reduction method. Our study aligns with Knauf et al. in that open reduction was not associated with higher complication rates post-operatively [8].

There is also conflicting published results regarding clinical outcomes based on implant choice. Our result contradicts Xie et al.’s results. They published intramedullary fixation which resulted in decreased failure rates and increased rates of re-operation compared to extramedullary fixation [7]. Additionally, Rahme et al. found intramedullary nailing to have a statistically significantly lower revision rate compared to fixed angle blade plating [15]. Lee et al. found no advantages to using an intramedullary nail versus dynamic condylar screw [16]. However, this study was performed in comminuted subtrochanteric fractures in patients with a mean age of 36.1 years. So, there are likely confounding factors that do not match our study. The available research is minimal in investigating outcomes comparing intramedullary nailing versus plating in the treatment of subtrochanteric femur fractures. There is some research available finding an intramedullary construct yields less post-operative pain and less re-operations compared to a sliding hip screw construct [10]. Another study investigated clinical outcomes in standard gamma nail fixations compared to Medoff sliding plate. This study found standard gamma nail fixations overall had lower complications post-operatively, including wound infection [11]. However, both studies were conducted in both trochanteric and subtrochanteric fractures and may not be generalizable to isolated subtrochanteric fractures. Beingessner et al. performed a case series in patients with closed subtrochanteric fractures all treated with open reduction and intramedullary nailing. They found all these patients to have successful osseous union, no wound complications, and no loss of reduction in post-operative radiographs [14]. Another retrospective analysis investigated patients treated with IMN for subtrochanteric femur fractures, where 10% resulted in non-union requiring an additional operation [17]. There are some studies available that investigate the use of various plate types in the treatment of subtrochanteric femur fractures. In summary, these studies show good fracture reduction, most cases achieved osseous union, and some resulted in soft tissue infection post-operatively [18–20]. None of the current available research directly compares the potential effects of intramedullary nail versus plate fixation on radiographic or clinical outcomes.

Our study revealed statistically significant increases in maximal cortical displacement on lateral imaging in closed reductions and intramedullary nail fixation. No differences in all other radiographic data were found when comparing reduction method or implant choice. The other radiographic data of interest were contralateral femoral neck-shaft angle (FNSA), FNSA, maximal cortical displacement on AP imaging, presence of varus or valgus on AP, presence of flexion or extension on lateral, and mRUST scores at 6 weeks, 3 months, 6 months, and 1 year. Beingessner et al. found no difference in coronal or sagittal deformity after open reduction and intramedullary nail fixation, which contradict our results [14]. Zhou et al. found no differences in radiographic healing during 6–12 month follow-up after closed or open reduction followed by intramedullary nailing of subtrochanteric femur fractures^21^.

Overall, our study demonstrates open and closed reduction and nail, plate, or a combination of both can yield good outcomes clinically and radiographically in the treatment of subtrochanteric femur fractures. Our study does have some limitations. Although our mean follow-up days for all patients was 132 days, 12 patients were completely lost to follow-up and had 0 clinical follow-up days. Additionally, given the retrospective nature of our study design, there were missing data points for some patients. This likely impacts our mRUST scoring and we obviously were not able to evaluate their post-operative clinical outcomes. Our study was performed only at one institution over nine years with strict inclusion criteria that likely filtered our sample size significantly. Even with the limitations, this study provides insightful, novel information to the currently published literature.

## Conclusion

Our study directly compares reduction methods and implant choices in the treatment of primary traumatic subtrochanteric femur fractures. Overall, most of our outcomes clinically and radiographically yielded no significant differences when comparing reduction method and implant choice. As such, specific fracture patterns and patient characteristics drive the above as expected. These findings are helpful and beneficial for surgeons when determining which reduction method or implant choice to use, as there is no mandatory or necessary approach, but rather excellent outcomes can be achieved with a variety of techniques.

## Data Availability

No datasets were generated or analysed during the current study.

## References

[1] Medda S, Reeves RA, Pilson H. Subtrochanteric Femur Fractures. In: *StatPearls*. StatPearls Publishing; 2024. Accessed April 25, 2024. http://www.ncbi.nlm.nih.gov/books/NBK507803/29939580

[2] Plumarom Y, Wilkinson BG, Willey MC, An Q, Marsh L, Karam MD (2021) Sensitivity and specificity of modified RUST score using clinical and radiographic findings as a gold standard. Bone Jt Open 2(10):796–805. 10.1302/2633-1462.210.BJO-2021-0071.R1 34587782 10.1302/2633-1462.210.BJO-2021-0071.R1 PMC8558446

[3] Urva M, Challa ST, Haonga BT et al (2022) Reliability of modified radiographic union score for tibia scores in the evaluation of femoral shaft fractures in a low-resource setting. J Am Acad Orthop Surg Glob Res Rev 6(5):e21.00211. 10.5435/JAAOSGlobal-D-21-00211 35605095 10.5435/JAAOSGlobal-D-21-00211 PMC9126518

[4] Mısır A, Yıldız Kİ, Kızkapan TB, Uzun E, Özçamdallı M, Oğuzkaya S (2021) Reliability of RUST and modified RUST scores for evaluation of union in pediatric and adult femoral shaft fractures. Acta Orthop Traumatol Turc 55(2):127–133. 10.5152/j.aott.2021.20074 33847574 10.5152/j.aott.2021.20074 PMC11229616

[5] Panteli M, Vun JSH, West RM, Howard AJ, Pountos I, Giannoudis PV (2022) Management of subtrochanteric femur fractures: is open reduction associated with poor outcomes? Eur J Trauma Emerg Surg 48(3):1759–1768. 10.1007/s00068-021-01834-6 34825927 10.1007/s00068-021-01834-6 PMC9192396

[6] Tahir M, Ahmed N, Faraz A, Shafiq H, Khan MN (2021) Comparison of open and closed nailing for femoral shaft fractures: a retrospective analysis. Cureus 13(6):e16030. 10.7759/cureus.16030 34336517 10.7759/cureus.16030 PMC8319164

[7] Xie H, Xie L, Wang J, Chen C, Zhang C, Zheng W (2019) Intramedullary versus extramedullary fixation for the treatment of subtrochanteric fracture: a systematic review and meta-analysis. Int J Surg 63:43–57. 10.1016/j.ijsu.2019.01.021 30735845 10.1016/j.ijsu.2019.01.021

[8] Knauf T, Eschbach D, Buecking B et al (2021) Open reduction in subtrochanteric femur fractures is not accompanied by a higher rate of complications. Medicina (B Aires) 57(7):659. 10.3390/medicina57070659 10.3390/medicina57070659 PMC8305416 34199013

[9] Yao X, Wu L, Li J et al (2020) Effectiveness analysis of closed or limited open reduction and intramedullary nail fixation in treatment of Seinsheimer type V subtrochanteric fracture. Zhongguo Xiu Fu Chong Jian Wai Ke Za Zhi Zhongguo Xiufu Chongjian Waike Zazhi Chin J Reparative Reconstr Surg 34(4):457–462. 10.7507/1002-1892.201908100 10.7507/1002-1892.201908100 PMC8171499 32291981

[10] Matre K, Havelin LI, Gjertsen JE, Vinje T, Espehaug B, Fevang JM (2013) Sliding hip screw versus IM nail in reverse oblique trochanteric and subtrochanteric fractures. A study of 2716 patients in the Norwegian Hip Fracture Register. Injury 44(6):735–742. 10.1016/j.injury.2012.12.010 23305689 10.1016/j.injury.2012.12.010

[11] Miedel R, Ponzer S, Törnkvist H, Söderqvist A, Tidermark J (2005) The standard gamma nail or the Medoff sliding plate for unstable trochanteric and subtrochanteric fractures. A randomised, controlled trial. J Bone Joint Surg Br 87(1):68–75 15686240

[12] Şensöz E, Cecen G (2023) Comparison of intramedullary and extramedullary fixation results in subtrochanteric femur fractures. Cureus 15(11):e49258. 10.7759/cureus.49258 38143672 10.7759/cureus.49258 PMC10746348

[13] Mirbolook A, Siavashi B, Jafarinezhad AE et al (2015) Subtrochanteric fractures: comparison of proximal femur locking plate and intramedullary locking nail fixation outcome. Indian J Surg 77(Suppl 3):795–798. 10.1007/s12262-013-1004-3 27011459 10.1007/s12262-013-1004-3 PMC4775634

[14] Beingessner DM, Scolaro JA, Orec RJ, Nork SE, Barei DP (2013) Open reduction and intramedullary stabilisation of subtrochanteric femur fractures: a retrospective study of 56 cases. Injury 44(12):1910–1915. 10.1016/j.injury.2013.08.013 24021583 10.1016/j.injury.2013.08.013

[15] Rahme DM, Harris IA (2007) Intramedullary nailing versus fixed angle blade plating for subtrochanteric femoral fractures: a prospective randomised controlled trial. J Orthop Surg Hong Kong 15(3):278–281. 10.1177/230949900701500306 18162669 10.1177/230949900701500306

[16] Lee PC, Hsieh PH, Yu SW, Shiao CW, Kao HK, Wu CC (2007) Biologic plating versus intramedullary nailing for comminuted subtrochanteric fractures in young adults: a prospective, randomized study of 66 cases. J Trauma 63(6):1283–1291. 10.1097/TA.0b013e3180f62f00 18212651 10.1097/TA.0b013e3180f62f00

[17] Pavelka T, SalÁŠek M (2020) Treatment of subtrochanteric fractures, our experience, complications. Acta Chir Orthop Traumatol Cech 87(4):259–267 32940221

[18] Chiu YC, Wu CH, Tsai KL, Jou IM, Tu YK, Ma CH (2022) External locking plate fixation for femoral subtrochanteric fractures. Geriatr Orthop Surg Rehabil 13:21514593221124416. 10.1177/21514593221124416 36081842 10.1177/21514593221124416 PMC9445469

[19] Gogna P, Mukhopadhyay R, Singh A et al (2015) Contralateral reversed distal femoral locking plate for fixation of subtrochanteric femoral fractures. Chin J Traumatol 18(5):279–283. 10.1016/j.cjtee.2015.11.002 26777711 10.1016/j.cjtee.2015.11.002

[20] Hu SJ, Zhang SM, Yu GR (2012) Treatment of femoral subtrochanteric fractures with proximal lateral femur locking plates. Acta Ortop Bras 20(6):329–333. 10.1590/S1413-78522012000600003 24453626 10.1590/S1413-78522012000600003 PMC3861956

[21] Zhou ZB, Chen S, Gao YS, Sun YQ, Zhang CQ, Jiang Y (2015) Subtrochanteric femur fracture treated by intramedullary fixation. Chin J Traumatol 18(6):336–341. 10.1016/j.cjtee.2015.11.011 26917024 10.1016/j.cjtee.2015.11.011

